# Management and Outcomes of Low-Grade Gliomas in Africa: A Scoping Review Protocol

**DOI:** 10.29337/ijsp.171

**Published:** 2022-02-02

**Authors:** Setthasorn Zhi Yang Ooi, Rosaline de Koning, Abdullah Egiz, David Ulrich Dalle, Moussa Denou, Marvin Richie Dongmo Tsopmene, Mehdi Khan, Régis Takoukam, Jay Kotecha, Dawin Sichimba, Yao Christian Hugues Dokponou, Ulrick Sidney Kanmounye, Nourou Dine Adeniran Bankole

**Affiliations:** 1 Research Department, Association of Future African Neurosurgeons, Yaounde, Cameroon; 2 Ibn Sina Teaching Hospital, Rabat Morocco/Mohammed V University of Rabat Morocco, Department of Neurosurgery, Rabat, Morocco

**Keywords:** low-grade gliomas, Africa, epidemiology, management, outcomes, scoping review, protocol

## Abstract

**Background:**

Over the last decade, many advancements have been made in the management of low-grade gliomas (LGGs). Overall survival outcomes are correlated with factors such as postoperative residual volumes and specific tumor biomolecular profiles such as IDH mutation status. It is unclear whether these advancements have benefited LGG patients in Africa. This scoping review protocol outlines how the authors will evaluate the epidemiology, presentations, management and outcomes of LGGs in Africa.

**Methods:**

MEDLINE, Embase and African Journals Online will be searched from database inception to date in order to identify the relevant studies. Patients of all ages with histologically and/or radiologically confirmed LGGs that were managed in an African country will be included. Surgical and chemoradiation management of LGG tumours will be considered. Original research, reviews, commentaries, editorials and case reports will be included.

**Results:**

Primary outcomes of the review will include LGG management, morbidity and mortality. Secondary outcomes include epidemiology and recurrence of LGGs.

**Discussion:**

This scoping review will be the first to evaluate the current landscape of LGG management and outcomes in Africa, highlighting pertinent themes that may be used to guide further research as well as health system strengthening efforts by policymakers and stakeholders.

**Scoping Review Registration:**

The protocol has been registered on the Open Science Framework (OSF; registration link: *https://doi.org/10.17605/OSF.IO/E732G*).

**Highlights:**

- LGGs account for 17% to 22% of total brain tumours and have a median survival time between 5.6 and 13.3 years.
- Despite many recent advancements in the management of LGGs, there is a paucity in the data within the African landscape.
- This scoping review will be the first to evaluate the current landscape of LGG management and outcomes in Africa, highlighting pertinent themes that may be used to guide further research and policymaking efforts.

## 1. Introduction

Low-grade gliomas (LGGs) are grade I and II primary brain tumors, according to the World Health Organization (WHO) [1]. LGGs include astrocytomas, oligodendrogliomas and oligoastrocytomas, with the latter being designated as Not Otherwise Specified (NOS) in the new 2016 WHO classification of central nervous system tumours [2]. They develop from glial cells and are characterized by a low proliferation index (median, 4.4 mm/yr) [3]. Nevertheless, they have a high potential of malignant transformation over time and can cause considerable morbidity and lead to death [4]. The annual global age-standardized incidence of primary malignant brain tumors is estimated at 3.7 and 2.6 per 100,000 males and females, respectively [5]. There is a marked increase of these rates in high-income countries (HICs) (men, 5.8 and women, 4.1 per 100,000) more than in low- and middle-income countries (LMICs) (men 3.0 and women 2.1 per 100,000) [5], however this could be due to an under-diagnosis of LGGs in LMICs. In Sub-Saharan Africa, brain tumors represent 0.14% of disability-adjusted life years (DALYs) and 0.17% of deaths, while in the Northern African and the Middle Eastern regions brain tumors cause 0.44% of DALYs and 0.62% of deaths [6]. LGGs represent between 17% to 22% of brain tumors (approximately 20 000 cases per year) and have a median survival time between 5.6 and 13.3 years [7].

Anecdotal evidence suggests that the management of patients with LGGs is challenging in Africa. Poor access to neuroimaging facilities leads to delays in diagnosis and referral [8]. Diagnosis and subsequent management decisions require the use of advanced and costly imaging modalities, which are not available in many resource limited settings. Though basic neuroimaging tools such as computerized tomography (CT) scan and magnetic resonance imaging (MRI) are being acquired widely, they are distributed unevenly, along with the already poor neurosurgical workforce [9]. Gross total resection is also a daunting task because of the absence of supportive technology. Moreover, high out-of-pocket costs and lack of financial risk protection limit access when those facilities are available [10]. In addition, infrastructural deficiencies limit service delivery. For example, only 10% of awake craniotomy LGG operations are done in optimal conditions [9], and there is a significant deficit in the literature on neurosurgical care for LGGs in Africa [11].

To our knowledge, there is no literature evaluating epidemiology, management and outcomes of patients with LGGs in Africa, hence necessitating the need for a scoping review.

## 2. Aims

*Primary aims*

1. Assess the treatment modality and management plan (i.e., chemotherapy, radiotherapy and intraoperative surgical adjuncts), and
2. Assess the clinical outcome of patients defined as rates of mortality, morbidity, and recurrence.

*Secondary aims*

1. Assess the epidemiology of LGGs in different African countries,
2. Assess the availability of diagnostic modalities such as histopathology and/or molecular pathology testing and neuroimaging (MRI, CT scan), and
3. Assess the availability of specialized workforce, infrastructure and financial risk protection.

## 3. Methods

The review will be conducted per the Arksey and O’Malley framework [12] and reported in accordance with the Preferred Reporting Items for Systematic Review and Meta-Analysis Extension for Scoping Reviews (PRISMA-ScR) guidelines [13]. A scoping review is chosen instead of a systematic review because the evidence relating to the epidemiology, presentation, management and outcomes of LGGs in Africa has not been comprehensively reviewed. Systematic reviews answer a focused research question with narrow parameters, strict endpoints, and eligibility criteria of the included studies defined at the outset, whereas scoping reviews can explore several questions in a broad sense. A scoping review is, therefore, more suitable for our investigation because it remains relatively unclear which specific questions should be asked and valuably addressed by a more precise systematic review and meta-analysis.

This protocol has been developed in accordance with the Preferred Reporting Items for Systematic Review and Meta-Analysis Protocols (PRISMA-P) guidelines [14]. The review was registered on Open Science Framework [15]. Protocol amendments will be updated and published alongside the scoping review results (*https://doi.org/10.17605/OSF.IO/E732G*).

### 3.1. Eligibility criteria

*Inclusion criteria*

Included will be any relevant article published in a peer-reviewed journal that discusses the epidemiology, presentation, management and outcomes of LGGs in Africa. Study types including journal articles, reviews, case reports, letters will be included. There will be no restrictions on the age of patients included nor the time period of publication. Publications in English and French languages will be considered.

*Exclusion criteria*

The following articles will be considered as outside the scope of this work and will be excluded:

- Do not include African patients or do not have disaggregated data about the African population,
- Do not discuss LGGs or do not have disaggregated data about LGGs,
- Do not discuss epidemiology, presentation, management or outcomes of patients with LGGs,
- Are neither written in English nor French, and
- Conference abstracts (due to the lack of in-depth information available).

### 3.2. Information sources

The databases to be searched include: MEDLINE, Embase and African Journals Online.

### 3.3. Search strategy

A search strategy has been developed to identify studies related to the treatment and/or outcomes of patients with LGGs in Africa. Synonyms relating to terms describing individual African countries, LGG, therapy, and outcome will be used (Supplementary Figure 1).

### 3.4. Data management

Data records will be downloaded from respective databases in comma-separated values (CSV) formatted files. They will then be imported into Rayyan [16] where deduplication, title and abstract screening, and full-text screening will take place. Further data extraction and quality assessment will be carried out on Microsoft Excel (Microsoft, Richmond, Virginia, USA).

### 3.5. Study selection

A calibration exercise will be carried out before title and abstract screening in order to ensure adequate understanding of the inclusion criteria by study screeners. Deduplication will be undertaken on Rayyan. Each study will then be screened using title and abstract, by two independent reviewers, against the pre-defined inclusion and exclusion criteria. Potentially eligible studies will be further screened for full-text review. Disagreements will be discussed amongst the reviewers and in case of no resolution, an appeal will be made to a senior author (USK or NDAB).

### 3.6. Data extraction

Full-text screened articles will be exported into a previously-made data extraction proforma on Microsoft Excel (Microsoft, Richmond, Virginia, USA). Data will be extracted on (i) study design, (ii) patient demographics, (iii) country of origin, (iv) tumour characteristics, (v) neuroimaging modality used, (vi) histopathology diagnosis, (vii) molecular pathology diagnosis, (viii) type of intervention, and (ix) outcomes of care. Data extraction will be performed in two stages, a pilot stage followed by a proper stage. The pilot stage will consist of having multiple authors, each going through the same 10 randomly selected articles to extract data. This is to assure the reliability of the proforma and that all participant authors were able to extract data accurately and homogeneously. Feedback from the pilot stage will inform any necessary changes to be made, upon discussion, in order to accurately capture the pertinent themes in the literature.

### 3.7. Risk of bias assessment

The purpose of this review is to produce a systematically conducted scoping review of the available literature to provide a comprehensive overview on the management and outcomes of LGGs in Africa, hence a formal bias assessment will not be conducted. Given the limited and heterogenous literature body, a formal bias risk assessment was deemed unnecessary for this emerging area of literature, as it will suffer from standard biases associated with new areas of clinical research.

### 3.8. Data synthesis

Study characteristics will be summarised using descriptive statistics and presented in a table. Data relating to study characteristics will be grouped into categories where appropriate. Categorisation might be based on type of LGG tumours, countries/regions within Africa or will be finalised in discussions with the team wherever necessary if notable differences emerge in study findings.

Pertinent characteristics of the study population will be analysed particularly relating to age, all which contribute to the risk of being diagnosed with LGG and more importantly the subsequent outcomes. Particular features of the tumour will be noted, in order to gauge all the factors that have contributed to tumour prognosis in study participants, such as those related to tumour biology (histology and molecular pathology), tumour location, solitary or multiple-lesion tumours, and tumour recurrence. The intraoperative adjuncts used during surgery and the extent of resection for these tumours will also be highlighted whenever applicable. Any notable surgical adjuncts used in improving the extent of resection will be reported. The treatment outcomes will be described: mortality rate, morbidity rate, and recurrence. An analysis of these outcomes will enable comparison and discussion of effectiveness of treatment techniques adopted in Africa with HICs and other LMICs.

## 4. Ethics and dissemination

This study will exclusively involve secondary data collection and no human participants will be involved in the design or dissemination of this research, hence ethical approval was not required. The results from this study will be disseminated through a peer-reviewed journal.

## 5. Limitations

There is an extensive amount of literature published in Arabic, Portuguese and Spanish that will not be addressed by this review.

## 6. Conclusion

The proposed scoping review aims to detail the current landscape of LGGs in Africa, highlighting parallels and differences among countries within the continent as well as with other countries of different income groups. This novel work will allow for a better understanding of the current situation in Africa, wherein important lessons can be drawn from the robust workforce environment and the use of advanced surgical adjuncts in HICs, and vice-versa from the ingenious thinking that is common in regions with limited resources. This would provide pertinent lessons to be learnt to improve patient outcomes and the quality of care of LGG management, especially given its poor prognosis of progressing to high-grade glioma and eventually death [4].

## Amendments

Any amendments to this protocol will be prospectively updated on Open Science Framework.

## Additional File

The additional file for this article can be found as follows:

10.29337/ijsp.171.s1 Supplementary Figure 1. Search strategy.
