# Supplementary material for: Management and Outcomes of Low-Grade Gliomas in Africa: A Scoping Review Protocol
Source: Int J Surg Protoc. 2022 Feb 2;26(1):1–6. doi: 10.29337/ijsp.171 (PMC8815446; doi:10.29337/ijsp.171)
Supplement: Supplementary Figure 1. — Search strategy. [file ijsp-26-1-171-s1.pdf]

Supplementary Figure 1

Search strategy

MEDLINE

	Query
01	“Developing Countries"[Mesh] OR Developing Countr*[tiab] OR “Least Developed Countries”[tiab] OR “Least Developed Country”[tiab] OR Less-Developed Countr*[tiab] OR Under-Developed Nations[tiab] OR Under-Developed Nation[tiab] OR Underdeveloped Nations[tiab] OR Underdeveloped Nation[tiab] OR Third-World Countr* [tiab] OR Third-World Nations[tiab] OR Third-World Nation[tiab] OR Under-Developed Countr*[tiab] OR Underdeveloped Countr*[tiab] OR Developing Nations[tiab] OR Developing Nation[tiab] OR Less-Developed Nations[tiab] OR Less-Developed Nation[tiab] OR “low and middle income countries”[tiab] OR low-to-middle-income countries[tiab] OR LMIC[tiab] OR Low-income countr*[tiab] OR “low-income economy”[tiab] OR “low-income economies”[tiab] OR “Low-income nation”[tiab] OR “Low-income nations”[tiab] OR “Low-Resource countries”[tiab] OR “Low-Resource nation”[tiab] OR “Low-Resource nations”[tiab] OR “Resource-limited countries”[tiab] OR “Resource-limited nation”[tiab] OR “Resource-limited nations”[tiab] OR “Resource-limited setting”[tiab] OR Poor countr*[tiab] OR “Ethiopia”[tiab] OR “Egypt”[tiab] OR “Democratic Republic of the Congo”[tiab] OR “Tanzania”[tiab] OR “South Africa”[tiab] OR “Kenya”[tiab] OR “Algeria”[tiab] OR “Sudan”[tiab] OR “Morocco”[tiab] OR “Uganda”[tiab] OR “Mozambique”[tiab] OR “Ghana”[tiab] OR “Angola”[tiab] OR “Somalia”[tiab] OR “Ivory Coast”[tiab] OR “Madagscar”[tiab] OR “Cameroon”[tiab] OR “Burkina Faso”[tiab] OR “Niger”[tiab] OR “Malawi”[tiab] OR “Zambia”[tiab] OR “Mali”[tiab] OR “Senegal”[tiab] OR “Zimbabwe”[tiab] OR “Chad”[tiab] OR “Tunisia”[tiab] OR “Guinea”[tiab] OR “Rwanda”[tiab] OR “Benin”[tiab] OR “Burundi”[tiab] OR “South Sudan”[tiab] OR “Eritrea”[tiab] OR “Sierra Leone”[tiab] OR “Togo”[tiab] OR “Libya”[tiab] OR “Central African Republic”[tiab] OR “Mauritania”[tiab] OR “Republic of the Congo”[tiab] OR “Liberia”[tiab] OR “Namibia”[tiab] OR “Lesotho”[tiab] OR “Gambia”[tiab] OR “Gabon”[tiab] OR “Guinea-Bissau”[tiab] OR “Mauritius”[tiab] OR “Equatorial Guinea”[tiab] OR “Eswatini”[tiab] OR “Djibouti”[tiab] OR “Comoros”[tiab] OR “Cape Verde”[tiab] OR “São Tomé and Príncipe”[tiab] OR “Seychelles”[tiab] OR “Ethiopia”[MeSH] OR “Egypt”[MeSH] OR “Democratic Republic of the

	Congo”[MeSH] OR “Tanzania”[MeSH] OR “South Africa”[MeSH] OR “Kenya”[MeSH] OR “Algeria”[MeSH] OR “Sudan”[MeSH] OR “Morocco”[MeSH] OR “Uganda”[MeSH] OR “Mozambique”[MeSH] OR “Ghana”[MeSH] OR “Angola”[MeSH] OR “Somalia”[MeSH] OR “Ivory Coast”[MeSH] OR “Madagscar”[MeSH] OR “Cameroon”[MeSH] OR “Burkina Faso”[MeSH]OR “Niger”[MeSH] OR “Malawi”[MeSH] OR “Zambia”[MeSH] OR “Mali”[MeSH] OR “Senegal”[MeSH] OR “Zimbabwe”[MeSH] OR “Chad”[MeSH] OR “Tunisia”[MeSH] OR “Guinea”[MeSH] OR “Rwanda”[MeSH] OR “Benin”[MeSH] OR “Burundi”[MeSH] OR “South Sudan”[MeSH] OR “Eritrea”[MeSH] OR “Sierra Leone”[MeSH] OR “Togo”[MeSH] OR “Libya”[MeSH] OR “Central African Republic”[MeSH] OR “Mauritania”[MeSH] OR “Republic of the Congo”[MeSH] OR “Liberia”[MeSH] OR “Namibia”[MeSH] OR “Lesotho”[MeSH] OR “Gambia”[MeSH] OR “Gabon”[MeSH] OR “Guinea-Bissau”[MeSH] OR “Mauritius”[MeSH] OR “Equatorial Guinea”[MeSH] OR “Eswatini”[MeSH] OR “Djibouti”[MeSH] OR “Comoros”[MeSH] OR “Cape Verde”[MeSH] OR “São Tomé and Príncipe”[MeSH] OR “Seychelles”[MeSH]
02	"low glioma"[tiab] OR "glioma"[MeSH] OR "glioma"[tiab] OR "gliomas"[MeSH] OR "gliomas"[tiab] OR "astrocytoma"[MeSH] OR "astrocytoma"[tiab] OR "astrocytomas"[tiab] OR "oligodendroglioma"[MeSH] OR "oligodendroglioma"[tiab] OR "oligodendrogliomas"[tiab] OR "oligoastrocytomas"[tiab] OR "oligodendroglioma"[MeSH] OR "oligoastrocytoma"[tiab]
03	"therapeutics"[MeSH] OR "therapeutics"[tiab] OR "treatments"[tiab] OR "therapy"[MeSH] OR "therapy"[tiab] OR "treatment"[tiab] OR "treatments"[tiab] OR "therapeutic"[tiab] OR "therapies"[tiab] OR "managment"[tiab] OR "management"[MeSH]
04	"prognosis"[MeSH] OR "prognosis"[tiab] OR "prognoses"[tiab] OR "prognostic"[tiab] OR "prognostical"[tiab] OR "prognostically"[tiab] OR "prognosticate"[tiab] OR "prognosticated"[tiab] OR "prognosticates"[tiab] OR "prognosticating"[tiab] OR "prognostication"[tiab] OR "prognostications"[tiab] OR "prognostics"[tiab] OR "prognoses"[tiab] OR "outcome"[tiab] OR "outcomes"[tiab] OR "outcomes"[MeSH]
05	(01) AND (02) AND (03) AND (04)

Embase

	Query
01	exp "Africa south of the Sahara"/ or exp South Africa/ or exp Africa/ or exp North Africa/ or exp Central Africa/ OR exp Cote d'Ivoire/ OR exp Ethiopia/ OR exp Egypt/ OR exp Democratic Republic Congo/ OR exp Tanzania/ OR exp South Africa/ OR exp Kenya/ OR exp Algeria/ OR exp Sudan/ OR exp Morocco/ OR exp Uganda/ OR exp Mozambique/ OR exp Ghana/ OR exp Angola/ OR exp Somalia/ OR exp Madagascar/ OR exp Cameroon/ OR exp Burkina Faso/ OR exp Niger/ OR xp Malawi/ OR exp Zambia/ OR exp Mali/ OR exp Senegal/ OR exp Zimbabwe/ OR exp Chad/ OR exp Tunisia/ OR exp Guinea/ OR exp Rwanda/ OR exp Benin/ OR exp Burundi/ OR exp South Sudan/ OR exp Eritrea/ OR exp Sierra Leone/ OR exp Togo/ OR exp Libya/ OR exp Central African Republic/ OR exp Mauritania/ OR exp Mauritania/ OR exp Congo/ OR exp Liberia/ OR exp Namibia/ OR exp Lesotho/ OR exp Gambia/ OR exp Gabon/ OR exp Guinea-Bissau/ OR exp Mauritius/ OR exp Equatorial Guinea/ OR exp Eswatini/ OR exp Djibouti/ OR exp Comoros/ OR exp Cape Verde/ OR exp Seychelles/ OR exp developing country/ OR Least Developed Countries.mp. OR (low and middle income countries).mp. [mp=title, abstract, heading word, drug trade name, original title, device manufacturer, drug manufacturer, device trade name, keyword, floating subheading word, candidate term word]
02	exp glioma/ OR astrocytoma.mp. or exp astrocytoma/ or exp pilocytic astrocytoma/ or exp anaplastic astrocytoma cell line/ or exp astrocytoma cell line/ or exp astrocytoma cell/ or exp subependymal giant cell astrocytoma/ OR exp oligodendroglioma cell line/ or exp oligodendroglioma/ OR oligoastrocytoma.mp.
03	treatment.mp. [mp=title, abstract, heading word, drug trade name, original title, device manufacturer, drug manufacturer, device trade name, keyword, floating subheading word, candidate term word] OR therapeutic.mp. or exp therapy/ OR management.mp.
04	exp treatment outcome/ or outcome.mp. or exp clinical outcome/ OR exp prognosis/ or exp cancer prognosis/ OR exp prognostic assessment/ or prognostic.mp.
05	(01) AND (02) AND (03) AND (04)
